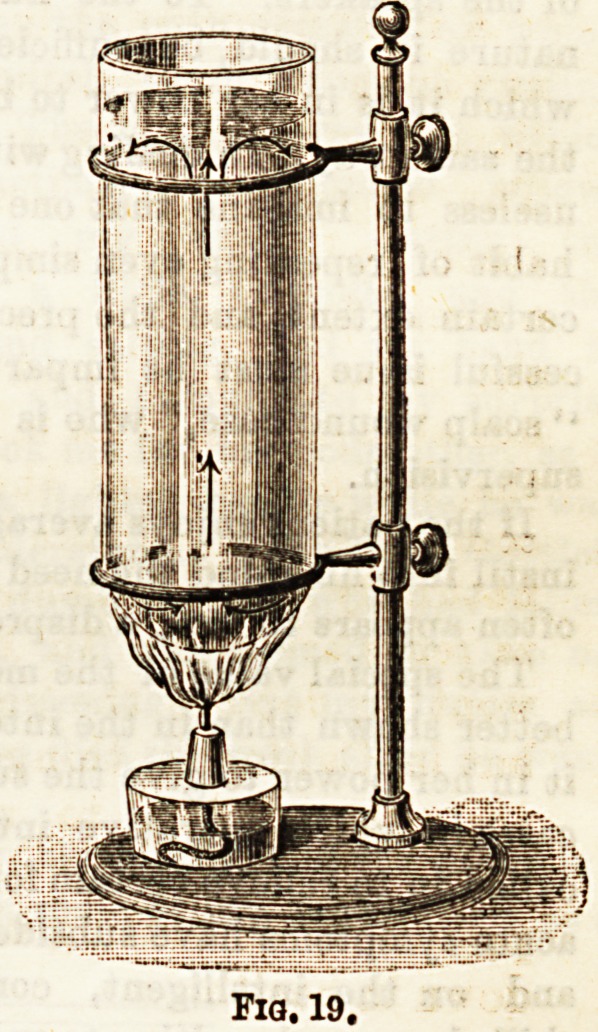# "The Hospital" Nursing Mirror

**Published:** 1896-05-30

**Authors:** 


### "The Hospital" Nursing Mirror.

Being the Extra Nursing Supplement of "The Hospital" Newspaper.

[Contributions for this Supplement should be addressed to the Editor, The Hospital, 428, Strand, London, W.C., and should have the word "Nursing" plainly written in left-hand top corner of the envelope.]

### News from the Nursing World.

### THE TROUBLES AT ROTHERHITHE.

At the last meeting of the St. Olave's and St. Thomas's Vestry Mr. Gridley called attention to the letter written by Miss Evans, matron of the St. Olave's Infirmary, to the *South London Press* with reference to her suspension by the Board of Guardians. (An extract from the letter in question appeared in these columns a fortnight ago.) He moved that "the guardians' attention be called to the statements of the matron, and that they be asked to send the vestry as soon as possible the whole of the case *pro* and *con*." The resolution was supported by Mr. E. Besley, who remarked that when himself on the Board of Guardians he "came to the conclusion that it was a grossly mismanaged institution," and after some rather angry remarks from Mr. Shand, one of the guardians, who held the opposite view, the resolution was carried.

### A "HOME OF REST" FOR NURSES.

At this time of year many nurses are contemplating arrangements for summer holidays, and to these the following extract from a letter from Miss Grey, lady superintendent of the Metropolitan Nursing Association, will perhaps be of use. "Nurses requiring rest and change of air will find comfortable quarters at a small Home of Rest, which has lately been opened in a pretty part of Hertfordshire. Sawbridgeworth may be reached from St. Pancras or Liverpool Street in an hour, and the single fare is only 2s. 3d. The village is picturesque and quiet, and there is a great variety of pleasant walks and excursions in the neighbourhood. The terms at the Home are most moderate. Address, Mrs. Osborne, The Limes, Sawbridgeworth."

### PREJUDICE IN JOURNALISM.

Our attention has been called to a statement in a contemporary to the effect that "at the Westminster "Police-court on Monday, the 18th instant, a man "complained to Mr. De Rutzen of his treatment in "No. 3 Ward of the Chelsea Infirmary, where he had "gone as a destitute sick person and a native of the "parish for rest and treatment." It appears on inquiry that No. 3 Ward of the Chelsea Infirmary is a female ward, and that the man in question was never an inmate of the Chelsea Infirmary at all. These facts the *Nursing Record*, which poses as a technical journal, should have known. It is to be regretted that people who never tire of asserting their claim to speak with authority on nursing matters should display such extraordinary ignorance of the difference between the workhouse and the poorlaw infirmaries of the metropolis. The man in question was, no doubt, an inmate of the workhouse, which, though under the same board of guardians, is under distinct administration and has nothing whatever to do with the Chelsea Poor Law Infirmary. We hope that in future our contemporary, before it presumes to pose as an authority, will see that both its editor and contributors are made to undergo a course of instruction in the poor law system of the metropolis. Prejudice in the Press against individuals is happily rare in this country, and when, as in the present case, it is accompanied by ignorance, justice will no doubt provide punishment by ensuring that criticisms on nursing matters from this quarter will be discredited in advance for the future.

### AN ENCOURAGING REPORT.

At a recent meeting of the Mile End Board of Guardians a most satisfactory report was read on the recent examination by Dr. Dean of the infirmary probationers. Dr. Dean stated that in comparison with the results of two former examinations he noticed "a very decided improvement in the written part of the examinations. The practical work was performed excellently on the two former occasions, so that the room for improvement in this respect was not large; the answers showed evidence of careful teaching." The medical superintendent and matron must be congratulated on this satisfactory result of a year's work.

### ZENANA MEDICAL MISSION.

A pleasant conversazione was held by invitation of Lord and Lady Kinnaird, on May 19th, at the Royal Institute of Painters in Water Colours, at which many friends of the Zenana Bible and Medical Mission were present. Lord Kinnaird and Sir Charles Elliott gave some account of the society's labours in India, the latter specially referring to the valuable services of Miss Mackinnon, M.D., at Patna, and the efficient working of the hospital there. Miss Gollock described some interesting experiences of her recent visit to India, and Mr. W. T. Paton, the hon. finance secretary, pleaded the cause of the society and its need for increased funds. Some £2,000 is required, in addition to the ordinary income, to enable the work to be carried on unhampered through the present year.

### THE TRUTH OF THE MATTER.

The committee of the Bristol Nurses' Training Institution, being "strongly urged by their medical advisers and the authorities of the training schools," have this year lengthened the period of hospital training for their nurses from one year to three, and a remark on the subject in their annual report deserves a moment's consideration. It is only voicing a feeling experienced by many who have to do with the requirements of private nursing to say that "there are some drawbacks to a prolonged residence in hospital considered as a preparation for private nursing" (besides the considerable expense involved); and the true reason for prolonging the period of probation at hospitals ought to be acknowledged. The fact is that the "third year," to which so much magic is imputed by some people, is exacted, not because it is needful for a private nurse to spend three years in the wards of a general hospital, but because, in order to keep up an efficient staff, the hospitals have to bind those whom they train to give back, as it were, a certain period of service, at the end of their probationary time, when they have become efficient. There is certainly reason in this from the hospital authorities' point of view, but it is rather hard on private nursing institutions, and adds to their expenses without any proportionate return benefit, that their probationers should be compelled to bind themselves for this long time in order to gain the necessary certificate, and run the danger of so getting into "ward ways" which have to be knocked out of them again before they can become valuable for private work. Be this as it may, the facts should be looked at and spoken of honestly. Practical medical men and nurses know well that for private and district work two years "in hospital" are ample, and so far as experience is concerned the third year had far better be devoted to training in the particular branch of nursing afterwards to be taken up. If the hospital training schools find it impossible to accept probationers for a shorter period than three years, it only remains to make the best of the matter, but let it be fully understood that this arrangement is made for the better nursing of the hospital patients, not for the benefit of the future private or district nurse.

### HOW THE CHILDREN HELP.

A cot, presented by Miss Maitland-Erskine, a late sister at Charing Cross Hospital to the Victoria Ward, is to be maintained, until such time as sufficient funds can be collected for permanent endowment, by the Mayfair branch of the Ministering Children's League, and its formal handing over to the hospital was the occasion of a little ceremony at Charing Cross the other day. The gift was presented by Lady Eleanor Harbord, president of the branch, to Mr. George Drummond, treasurer of the hospital, and a short dedicatory service was read by the Chaplain, several members of the staff and committee being present.

### LIFE IN CALIFORNIA.

A correspondent in Los Angeles, California, gives anything but an encouraging account of the conditions of private nursing in that quarter of the world. The wages are much lower than they were a few years since, and, although a good nurse can still get £2 to £3 a week, for that she has "to work night and day until she is completely tired out." Apparently, too, it is not always as easy as might be wished finally to obtain the fee earned, and the heavy strain when at work compels nurses to take long rests between cases, which naturally means considerable financial loss, especially as rents are high. In England people are nowadays wont to deplore the general "high pressure" of life, but in California things are worse; men and women are alike victims to nervous excitement, and the "hustle" for money getting, in conjunction with a peculiarly enervating climate, leads to many a breakdown in health.

### NURSES' PENSIONS.

In "The New System of Medicine," edited by Professor Clifford Allbutt, the first volume of which has just been issued, we notice an admirable article on nursing by Miss Amy Hughes. In it the following passage occurs: "Those interested in nurses as a class may give material help to individuals by advising and helping them in habits of thrift. The Royal Pension Fund for trained nurses and similar schemes offer unusual advantages for old-age pensions, sick pay, &c., and grateful patients may do much for the nurses by helping them to become members of such associations." These are sentiments which, finding a place in a book of such authority, will, we hope, often be borne in mind by medical men when asked by grateful patients how they can most appropriately express their sense of the good services rendered to them by a devoted nurse.

### PROGRESS IN INDIA.

A new hospital was opened at Gaya, in the Bengal Presidency, in March last, by Lady Elgin, wife of the Viceroy of India. The building is called after her name, and has been erected at a cost of over 40,000 rupees, entirely subscribed by local native generosity. Of this sum one native Babu gave 10,000 rupees to build the main Hindu ward, and the Rajah of Muksudpur has since offered the sum of 25,000 rupees for the establishment of "cottage" wards, to bear the names of Lady Elgin and Lady Elliott.

### SHORT ITEMS.

A "Nursing Exhibition," arranged by Mrs. Bedford Fenwick, is announced to take place at St. Martin's Town Hall from June 1st to June 13th. In connection with the exhibition papers on nursing questions will be read in the lecture room on the ground floor of the hall on June 3rd, 4th, and 5th by Mrs. Bedford Fenwick, Miss Isla Steward, Miss Mollett, and others. — At a largely-attended meeting of subscribers to the Queen Victoria Jubilee Nurse Fund held the other day at Ruthin Castle, the Bishop of St. Asaph, who was present, advocated guardians of the poor supporting district nursing associations by grants, as they had the power to do. There could, he thought, be no better way of carrying out their duty of helping the poor.—Dr. Sibbald, Commissioner in Lunacy, has recently made a most favourable report on the system of nursing at the Fife and Kinross Asylum, and congratulated the district board and Dr. Turnbull on an important step in the improvement of asylum management.—Sister Amy Evans, who is retiring from the post of lady superintendent of the Barry Docks Nursing Association and Accident Hospital, has addressed a letter to the editor of the *Barry Dock News* thanking him for "the constant kindness and exceptional help received . . . during five happy years of work" through his paper.—The Duchess of Teck has consented to become a vice-president of the Zenana Medical College.—A syndicate has been appointed by the Council of the Senate of Cambridge to consider the question of degrees for women, which will come before the Senate on June 4th.—At the Hospital for Sick Children, Great Ormond Street, some of the small convalescents enjoyed the treat of going for a drive on Whit Monday, by way of a Bank Holiday festivity.—The committee of the Paddington Green Children's Hospital have received a donation of £1,000 under the will of the late Mrs. Juliana Josephine McAlpine, for the endowment of a cot, to be named the "Margaret Charlotte McAlpine Cot."—On August 6th Madame Adelina Patti will give a grand morning concert at Swansea in aid of the Swansea Hospital and the poor of the district surrounding Craig-y-nos, the Welsh home of the great singer.

### Hygiene: For Nurses.

By John Glaister, M.D., F.F.P.S.G., D.P.H.Camb., Professor of Forensic Medicine and Public Health, St. Mungo's College, Glasgow, &c.

### VIII.—HEAT IN RELATION TO HEALTH.—MODES OF HEAT PROPAGATION.

Humboldt, the famous naturalist, in summing up the characteristics which differentiate man from the lower animals, declared that man was the only animal that cooked his food. If he had broadened the statement by saying also that man was the only animal that generated artificial heat for the purpose of warming his body, he would have made it even more forcible. On our planet the distribution of heat and cold is very unequal. The warmth of the Equator meets the converse of the frigidity at the Poles. Obviously, therefore, the incidence of man's position on the globe will affect the necessity which arises for the generation of artificial heat for the purpcse of maintaining the body warmth. The conservation of the heat of the body by artificial means at the Equator is less urgent than it is at the Poles; and between these two points—the extremes—the necessity of artificial heat becomes the more acute as is the distance from the warmer point, and as is the season of the year.

Apart from his food, man conserves the heat of his body in three ways, viz.: (1) By the erection of a shelter wherein to protect himself from the rudeness of the elements; (2) by some form of bodily covering; and (3) by the artificial generation of fire. These apply to man at any point of the earth's surface. The condition and character of the two former are determined by the local clemency or inclemency of weather, and he utilises the fire which cooks his food to warm his body.

Fig.17 illustrates reflection of radiant heat. A, body on which ray D strikes; O is point of absorption; B and E are angles of reflection.

The sum total of heat in the universe is always the same, varying nothing, never more, never less. It may be greater in amount in one place at any given time, or less at another, but such is the indestructibility of heat, energy, that the total amount never varies. Heat is not matter, but a form of energy. It therefore cannot be weighed. A vessel containing water at 60 deg. Fahr. will weigh precisely the same when the temperature is raised to 180 deg. Heat is generated simply by the commotion of the intimate particles of matter, which commotion may be set up in various ways—by rubbing a metal button on one's sleeve, or two pieces of dried wood together, or in water in a vessel over a flame, or in a vessel of water exposed to the direct rays of the sun. The human body is a machine in which heat is generated by the combustion of the food swallowed, and by the different chemical process which go on within it, much in the same way as in a steam-engine heat is generated in the water in its boiler by the combustion of coal. The temperature of the body in health is 98·5 deg. Fahr., and of the blood 100 deg. Fahr. These temperatures vary but within the slightest limits, whether at the Equator or the Poles. Man is, therefore, called a warm-blooded animal, since he retains his normal heat in the most varying environment. Other animals—as fishes and reptiles—are called cold-blooded, because their bodily temperatures accommodate themselves to the temperature of their environment, although, even for them, there are limits of heat and cold which are incompatible with life. In temperatures below the normal body heat, the human body parts with its heat, and it becomes essential, therefore, that artificial means must be adopted to prevent undue loss. The terms *amount* and *intensity* of heat are often used, but they do not mean the same thing. In a cupful and a kettle of water, each at 212 deg. Fahr., the intensity of heat will be identical, but the amount of heat in the latter will be proportionately larger, as is the relative quantity of water present in each. What is meant by temperature? It is the condition in which a body is in respect of hotness at any given time. The term temperature implies certain fixed points of heat and cold—some standard of heat measurement; for, after all, bodily sensations are but delusive guides. There are two fixed points of cold and heat with which all are acquainted, viz., when water freezes and when water boils. From these points an instrument has been made whereby we gauge heat amounts or temperatures, called a thermometer or heat-measurer. Of this instrument something will be said in another chapter.

Fig. 18.

Modes of Heat Propagation.—Whatever the source of heat, the heat so liberated flows towards the colder body, which it warms. If we could imagine a certain fixed amount of heat set free in an air-tight room the effect would be that the colder bodies in the room would be warmed until each attained the same uniform temperature. This is what happens in nature.

But we distinguish as to the mode by which heat is propagated through different bodies or media. There are three such modes, viz., radiation, convection, and conduction. Radiation is most common in aerial or gaseous bodies, convection in liquids, and conduction in solids. Radiation occurs in a vacuum as well as in air, and if air contained no particles the heat would pass through without warming it. The presence of particulate matter, however, causes a slight rise of temperature. Radiant heat rays are like light rays. They are propagated in all directions equally, and in right lines, and like light rays may be intercepted by a screen. Radiant heat is associated with luminosity. A lady shades her face from the sun by a parasol, or her furniture from the effects of heat by a screen. The intensity of radiant heat is in direct proportion to the temperature of its source, and inversely as the square of the distance from the source—that is, a body twice the distance from the source will only receive one-half, and one four times the distance one-fourth the heat of one at the source. When radiant heat falls upon an object, part is absorbed, part reflected, the respective amounts of each depending on (1) the material composing the body, (2) its surface-character, and (3) its colour.

Fig. 19.

Rough bodies absorb more heat than smooth, dark-coloured more than shining bodies. For this reason, outsides of kettles are rough and black, and engine fittings which require to be handled are kept clean and shining. Black clothes absorb more heat than they reflect; white clothes the reverse.

Convection is peculiar to liquids. A kettle boils best when placed on the fire, not when the fire is placed on the kettle. Fig. 18 illustrates this point. The water in a test-tube may be made to boil in its upper part, while its lower part will remain quite cold. In a kettle placed on the fire what happens is this: The layer of water next the fire first becomes heated; it therefore becomes lighter than the superposed layers, and rises to the top of the vessel; the layer which takes its place next becomes warmed and rises; and so on, till the whole column of water becomes of uniform temperature, *i.e.*, till it boils, as in Fig. 19.

### Trained Nurses' Clinic.

### VI.—THE NURSING OF HEAD CASES.

To the trained nurse few patients present more interesting features or call for more constant attention than those which we have ventured to summarise under the somewhat general term "Head Cases."

From the "Saturday night and Bank Holiday scalp wound" to the critical, obscure affection of the brain a wide field of experience comes under observation.

The scalp wound, whether trivial or extensive, whether simple or complicated by other injuries, demands attention and watchfulness to which the patient often consents with reluctance. In the case of out-patients at a hospital or dispensary, the nurse's chief responsibility (after she has assisted at the dressing of the wound) consists in endorsing and emphasising the surgeon's orders. She has facilities for doing this in her intercourse with the patient's friends, who are often disposed to listen to her friendly warnings, and will give them a certain amount of attention.

At any rate, it is assuredly part of the duty of the out-patient nurse to help the doctor's treatment by impressing upon all with whom she is brought in contact, the supreme importance of literal obedience to medical directions.

It may not be amiss to remark upon the fact that in dealing with persons of limited education it is always desirable to reiterate directions and cautions. No doubt this necessity is largely due to the custom which prevails amongst themselves of repeating each assertion not once but many times over.

In listening to the conversation of anyone possessed of narrow intellectual capacity this characteristic is exhibited to a striking extent. The repetition of surmises, incidents, or anticipations may become extremely wearisome, but it must be regarded as a valuable index to the habit of mind of the speakers. To the nurse who is a student of human nature it should be sufficiently obvious that the teaching which it is in her power to bestow should be also repeated in the same way. In dealing with this class of patients it is quite useless to imagine that one warning is enough; their own habit of repeating even simple facts must be imitated to a certain extent, and the precautions needful to ensure a successful issue must be imparted afresh at each visit of the "scalp wound case," who is not under constant professional supervision.

If the patient enjoys average health it is always difficult to instil into his mind the need for incessant prudence for, what often appears to him, a disproportionately long period.

The special value of the modern trained nurse is nowhere better shown than in the intelligent assistance which she has it in her power to give the surgeon in the after care of the cases in which operative interference has been successfully called in to relieve certain forms of brain diseases. After the acnte symptoms have subsided much still remains to be done, and on the intelligent, conscientious watchfulness of the skilled nurse the ultimate success of the surgeon is often, to some extent, dependent. Hence it is found that the latter shows considerable hesitation in engaging the services of a woman of whose previous work he has no personal acquaintance, whilst he generously acknowledges his obligation to those nurses who have already proved themselves worthy of trust under critical circumstances.

If the precautions and responsibilities attendant on the nursing of brain cases are serious, the reward for this work is correspondingly great, and few triumphs exceed that of the trained nurse who has assisted to "pull through a critical surgical or medical head case." If the patient is restored to health of body and mind after an accident or illness which threatened to affect his brain permanently the nurse may well rejoice. She has, besides carrying out orders, to exercise much discretion in her relations with those who are near of kin to the sufferer, for they not only ply her with questions, the answering of which is quite outside her own province, but they also desire to instruct her in a smattering of knowledge which they have acquired through some form of popular literature.

This is not an accompaniment of the treatment of brain disease only, but it is one which creates a difficulty, especially felt by private nurses in charge of these important cases.

The triumphs of modern surgery have introduced a measure of hopefulness in the lay mind to which is often superadded embellishments of imagination which cause the nurse doubts as to whether or not she should trouble to combat many marvellous assertions. It is often difficult to decide if silence or speech will best serve the interests of the patient, and it is a question which can only be settled on the merits of each special case. Keeping in view the supreme duty of loyalty to the doctor, combined with consideration of the patient, a nurse can hardly fail to hold the confidence of both, even if she has sometimes to discredit the stories of miraculous cures from impossible treatment, to which members of the household give utterance. "Head cases" include other than acute illnesses, but they all need accurate observation and infinite patience. A child with symptoms of meningitis may require the best nursing in the worst possible surroundings. It often seems hopeless for the nurse to secure absolute quiet, perfect ventilation, &c., for her charge, but the training which has taught her that these are essential has also developed in her the ability to face difficulties of environment which may be distinctly harmful to the sufferer, whose abnormal sensativeness to light and sound have been mistaken for unconsciousness or delirium by well-intentioned but ignorant friends.

### Minor Appointments.

Queen Charlotte's Lying-in Hospital.—Miss Marian Bose and Miss Elizabeth Hamilton have been appointed Ward Sister and Sister-Midwife, respectively, at this hospital. We have been unable to obtain particulars of previous training and appointments.

Suffolk General Hospital.—Miss Annie Bartlett has been appointed sister at this hospital. She was trained at the Devon and Exeter Hospital, and at the Lying-in Hospital, York Road. Miss Bartlett has had some experience in district work, and was for three years sister at the Poplar Hospital for Accidents.

Taunton and Somerset Hospital, Taunton.—Miss Robinson and Miss Roche have been appointed day ward sisters at this hospital. Miss Robinson was trained at Melbourne, afterwards holding the post of surgical ward sister at the General Hospital, Birmingham; and Miss Roche received her training at the London Hospital, where she afterwards worked as staff nurse. Miss Creagh has been appointed night sister. She has been promoted from the staff of the Taunton Hospital, where she trained.

### On Certain Aspects of the Nursing Question as Seen in England and Germany.

By a Certificated Midwife.

### VIII.—PROFESSORS AND PUPILS.

"When one goes to Rome one must do as the Romans," says the old proverb; and thus, though I considered that the manners of our learned doctor required mending, I accommodated myself to them, as I did to the police surveillance, and the curiosity of the postmen. "It is the custom of the country," said I when, returning to my lodging at the late hour of nine p.m., a policeman asked me where I was going at this time of night. "It is the custom of the country," said I again when the postman meeting me in the street with a letter in his hand would give it to me with the sympathetic remark that he hoped "all goes well with the Fraulein's Schatz?" or, "What news from England to-day?" And once more I said "It is the custom of the country," when the professors stormed and the pupils trembled. It is all very well in Germany, but how would it answer in England? And the problem is, can we get this first-rate teaching without surrendering many things which we hold dear? Would this sort of thing go down in an English hospital? One day a birth history had to be related in the ward, and a nervous scholar hesitated in beginning, hoping, I think, for some leading question from the doctor. "Why do you stand there like a stock or stone?" were his encouraging words, "if we wanted a statue to adorn our walls we should look out for something handsomer!" Thus adjured the trembling woman began. I do not say that with us she would have walked straight out of the ward, because such a scene would be impossible in England. Another time when at Vienna our much-respected professor was vituperating the female intellect in general and the class of pupils before him in particular; he was interrupted in his remarks by a loud fit of sneezing. The effect was ludicrous, when, rising to their feet in a body, these patient and polite Austrians cried out with one voice, "*Ihr Wohlsein Herr Professor!*" An American lady doctor who attended these classes with me buried her face in her pocket-handkerchief; I kept as composed a countenance as I could; needless to say, we both sat still in our places and bore the certain reflection on our want of manners. "*Bitte, bitte,*" replied the Professor, and resumed his remarks. One cannot judge other countries by our own standard. And this I will say for our good doctor at Stuttgart, that he made ample amends for all his harshness and rudeness when he gave his final address to the scholars at the close of the term. "You have thought me *streng*," said he (and the word is untranslatable, so I leave it in the original), after giving them a most admirable *résumé* of the duties and responsibilities of their calling. "You have often thought me *streng*, nay brutal perhaps"—"No, no, Herr Professor," was the cry that interrupted him. "Yes, it is true I have been *streng*. I have been harsh perhaps; I know it my children, but it was with an object in view. I have a heavy responsibility laid on me also, by the State and by God. It is my duty to turn you out as competent midwives, and the time allowed me for doing it in is short, and you are stupid, very stupid." "Yes, yes, Herr Professor, we are!" "Yes, you are stupid and ignorant, and you are only weak women, but you are called to the practice of a noble and difficult profession. Look at the mothers who put their lives in your hands; look at the infants—the future defenders of our great Fatherland—whose first cries appeal to you for help! Many a time, when you are in doubt what to do, many and many a time when some great emergency presents itself, my instructions will come back to you across the intervening years with all the force with which I fling them at you. When I have been hardest on you, my children, is when I have pictured to myself what would be the result if you made a mistake, or forgot your orders, and some poor mother's life was the forfeit of your ignorance. It is because I would spare you remorse at failure, because I value the mothers and wives of our great people that I hammer my words into your brain. Each cross word has been like a nail to fix some precept into your memory; each book I have flung at you will prevent some blunder; each curse will prove itself a blessing in disguise. And now, my scholars, go forth to your work, and let me, when I lie down to die, have the honour and pleasure of remembering that I have sent out a band of midwives who are among the most honourable and the most efficient in this great and glorious land of Germany!" And with that the good doctor held out his hands as it were in blessing, and the women swarmed round him with sobs and thanks. "God bless you, Herr Doctor," they cried, "we shall always remember your words." Some kissed his hands, others less effusive pressed them; all wept, including the worthy professor. As for me, I stood aside in contemplation of a scene wholly new to me, and pondered on many things. I heard, however, afterwards, some comments on the coldness of the English nature. Frau Schreivogel openly expressed her surprise that I had neither tears nor embraces at command. She had forgotten, I think, all her supplications to heaven on behalf of the *Herr Professor*; but, certainly, his heart was soft enough on that occasion. I make no comment on either his hardness or his softness; but while I record my personal gratitude to him, and my admiration of his most excellent teaching, I also say to myself in a whisper, "How would it do in England?"

One thing more I want to mention before I end these articles on the midwifery schools of Germany, and this is one which we should do well to copy. All the infants born in them are baptised before the mothers leave. In fact, every child born during the week is christened on the following Sunday afternoon. The Protestant minister and the Catholic priest both attend at the hospital, and all decent ceremony is observed; and friends or relations of the mothers come to look on or stand as sponsors—the only occasion on which they are allowed in the hospital, there being no visiting permitted to the patients. The pupils carry the babies laid on a white and sometimes lace-trimmed pillow. I remember that I held my charge during the whole service as he was a little Catholic, and the priest, with his anointing and salting, had no hand to spare for taking the child; and I found to my surprise afterwards that the priest took me for his godmother, as I was so obliging as to hold a lighted candle while he was saying something I did not understand. Poor little Gottlob was the child of a deaf and dumb mother who had fallen under my care to be nursed, and she seemed to have no friends to come to the christening. But my duties as sponsor were not lasting, for he died the week after he went out of the hospital, I heard.

So good-bye to the professor, the pupils, and the patients; and next week I will tell you some of my experiences as a typhoid case.

### Wants and Workers.

Nurse Jones, 31, Chester Street, S.W., is anxious to know if any institution or any charitable person would undertake the care of a young woman subject to epileptic fits. A lady would pay a small sum for her. [Has Nurse Jones applied to Lady Meath's Home of Comfort, Godalming?—Ed. *T.H.*]

### Lying-in Hospitals and Treatment by Midwives.

A careful consideration of the difficulties which have arisen in connection with the Ladies' Charity and Lying-in Hospital at Liverpool will show that to this question, as to almost every other, there are two sides, each of which seems fairly plausible from its own point of view. From the doctors' standpoint it is perfectly clear that the position taken up by the president and many of the subscribers to this charity is not only insulting to the medical profession but distinctly injurious to the interests of the patients; and that it is in a way a false pretence for an institution to appeal to the public for funds in the character of a hospital when in fact the patients admitted to it have no security that they will come under the care of properly qualified practitioners.

It cannot be denied, however, that the other side have a good deal to say for the position they take up. They say, This is not a hospital in the ordinary medical sense of the term. It is a lying-in charity, and lying-in is a normal process, not requiring a doctor at all; and they point out that whenever anything abnormal occurs they are quite ready to send for professional help. Their really important contention, however, is that, according to the rules of the institution, its object is defined (rule 2) as being "To provide poor married women with the assistance of trained midwives during their confinement, and when requisite of surgeon-accoucheurs."

It is clear also that there is considerable suspicion lurking in the minds of some of the subscribers that the medical staff want to utilise the clinical material for the teaching of medical students as opposed to midwives, and it does not seem at all improbable that this suspicion may be at the root of a good deal of the difficulty.

We may admit at once that there is a good deal to be said for the contention that the original aim of the charity was to provide midwives rather than doctors, and we think that we see underlying the whole difference the idea that parturition is a process in which women should be left to women. It is a feeling in which we have a certain sympathy, and no doubt there is much to be said for it. Nevertheless, we are bound to say that the doctors' view is the safe one. In this, as in many other hospital difficulties, the possession of a good and capable executive officer often glosses over and hides from the view of the committee the badness of a system. It is quite clear that in this case the committee repose great trust in their matron midwife, and against this we have nothing to say. But however good an individual officer may be, it is never a wise thing to allow the welfare of an institution to depend on the chance of obtaining a succession of paragons, and it will always be found that the permanent utility of a hospital will be better secured by so framing its rules as to attract to its service a staff of the highest talent than by throwing itself into the hands of an individual, however indispensable he or she may appear at the time.

Good as the results recently obtained may be, we understand that they were not always so, and we feel sure that the patients are likely to obtain a greater average security by throwing the responsibility for their treatment, from beginning to end, on a well-chosen and properly qualified staff than by entrusting it to any individual.

But, besides the patients, the midwives who get their training at this hospital must also be thought of. In midwifery, as in everything else, the rule holds good that the best teaching is to be had from those who teach out of a large store of knowledge rather than from those who teach merely just what they know; and we cannot but think that at the present day, when midwives are asking for themselves an assured status, it is a backward step to deprive their training school of the teaching of fully-qualified practitioners, even though that teaching depends, as it must usually do, upon the demonstration of ordinary cases.

In any case, an institution posing as a hospital should have a fully-qualified staff, whose advice and help, directly or indirectly rendered, should be open to every patient.

It should not be forgotten also that in the hundred years which have elapsed since this institution was founded, and even in the more than fifty years which have passed since the hospital was built, many things have changed, that old rules cannot be expected to take cognisance of modern conditions, and that if people are so very suspicious of the male element, and so very anxious that women should be attended only by persons of their own sex, it is not necessary as it was then to rely on midwives only, for there should be no difficulty now in obtaining a staff of fully qualified medical women.

It is to be feared, however, that the objection in reality is not so much to men as to science, and here we come to the important point which the people of Liverpool should definitely understand. No hospital has a right to offer to its patients less than the fullest measure of the advantages to be derived from modern scientific treatment. Whether, then, the staff consist of men or women, its members should be fully qualified, and should have full control over the treatment of every patient.

### How Small-pox is Nursed in Gloucester.

In the *Gloucester Journal* for May 16th appeared a full reprint of an article published in the Hospital Nursing Supplement for that week under the above heading, with an editorial comment thereon as follows:—

We direct our readers' attention to the extract from The Hospital, given in another column, arraigning the management of the small-pox hospitals. We have previously had knowledge of the grave charges made under this head, and for that reason we pressed for the official publication of the report which Dr. Brooke, recently appointed to the superintendence of the hospital, made to the sanitary committee a fortnight ago, with such remarks as the authority had to offer upon it. This duty cannot be any longer avoided, for the indictment of the administration now made in a medical journal of repute is so terrible that it cannot be ignored. The honour of the city demands that official cognisance should be taken of a series of charges circulated largely among the medical fraternity of the kingdom, and given to the world generally. It is so serious in every respect that the Sanitary Committee must deal with it in a comprehensive and authoritative manner. That public faith in the administration of the hospital has been gravely shaken cannot be questioned, for the reluctance of the attacked to go to the hospital has been one of the most notorious facts of the situation. If confidence is to be restored it is necessary that the charges now printed should be refuted if that be possible, or, if not, a solemn assurance given to the public that the admitted evils have been rectified.

This is a practical admission that the condition of affairs at the hospitals has been no secret in the city, and, in fact, many people must be well aware that matters were rather under than over stated by our correspondent. "The honour of the city" does certainly demand that Dr. Brooke's report shall be immediately made public, and its official publication should be insisted upon by the public of Gloucester without an hour's delay. Then it will be seen if adequate steps are now being taken to assist Dr. Brooke and his nursing staff to deal properly with the sick under their care.

At a meeting of the Gloucester City Council on the 19th inst., Dr. Campbell, Medical Officer of Health to the Gloucester Urban Sanitary Authority, reported under date 18th inst. as follows:

"That during the past fortnight a steady decline is to be observed in the smallpox epidemic, and I think this diminution will be maintained. The type of disease is also milder. Very few really bad cases have shown themselves during the timed named above.

"The city is almost free from any other infectious disease, and it is in an exceptionally satisfactory state in that respect; indeed, the number of cases other than smallpox for the past twelve months has been smaller than is usual in towns of this size."

### Holidays and Health.

[Readers of THE HOSPITAL in need of information about health resorts at home or abroad, or desirous of aid in forming holiday plans, are invited to send queries to EDITOR, 428, Strand, W.C. (marked "Travel" on outside of envelope), which will be answered under this section.]

### NORTH WALES.

NORTH WALES offers exceptional delights and advantages to the holiday maker, nurse or otherwise, whose object is to make the most, from the point of view of pleasure and health, of a short rest from a daily round of more or less hard work. There are obvious reasons why, for nurses who have to make such judicious use of their fortnight or three weeks as to brace them up for another year's labour, it is a great thing initially to avoid a very long journey, even if the holiday purse, which is not always as elastic as might be wished, would suffice for going far afield. The journey to almost any part of North Wales can be accomplished in six or seven hours, allowing, say, an hour or so for eccentricities on the part of the Cambrian railways, which take life in a singularly calm and unhurried way. And for the expense of the journey, thanks to the tourist tickets now issued by the London and North-Western Railway Company, the sum of under £2 will provide a return ticket to some of the most lovely spots in that most lovely country. The Great Western Railway also issues cheap tickets, and thus if desired is provided a choice of routes which is a considerable convenience. Going by Chester and Bangor, a return viâ Shrewsbury may be accomplished. On the tourist ticket, too, as not everyone seems to comprehend, the journey can be broken anywhere *en route*; and if untrammelled by much luggage, &c., an enjoyable and varied little tour can be accomplished at comparatively small expense.

During August, of course, lodgings are hard to find and expensive—very; but the "season" in Wales is a short one, and in the early months of the year, when the country is simply perfect, or in September and October, very reasonable terms are the rule, and exceptional ones would in many cases be made for nurses. During the winter, too, there are many sheltered spots on the coast and elsewhere well worth the journey north, and combining sea and mountain, with an absence of east wind, which makes them, even in cold weather, pleasant for holiday resort.

Railway and coach have made the most remote places accessible, and for good walkers the region of Snowdonia is ideal. An excellent centre for expeditions is Criccieth, on the northern coast of Cardigan Bay, an ancient fishing village not yet spoilt by trippers, in spite of the growing number of lodging-houses. At least, there is no pier with its attendant horrors, and niggers are almost unknown. It is a very quaint little town, divided in two by its Castle, of British origin, perched on a steep conical rock rising straight from the sea, and round which it is only possible to walk dryshod about twice in the year. Here is a glorious view over Tremadoc Bay, as the northernmost end of Cardigan Bay is called. To the right lies the Carnarvon Peninsula, terminating in Bardsley Island, the retreat, it is said, of 20,000 Christian refugees in the seventh century; on the other hand point beyond point of land stretches out into the far blue distance, with Aberystwith almost in front, and Barmouth and Harlech with its noble castle quite to the left, the Merioneth mountains (Cader Idris a faint peak only to be discerned in a very clear atmosphere) rising behind; quite to the rear, sharply defined, Snowdon; and nearer, imposing Moel Heborg, Moelwyn, and Moel-y-geist, the latter called by English visitors the Duke of Wellington, from the fancied resemblance to the profile of that great man in the outline of its peak.

Snowdon may be reached by more ways than one. The pleasantest is by driving to Beddgelert, eleven miles off, from whence the ascent is easily made. Beddgelert itself would be a charming spot to stay at for those who prefer mountain scenery pure and simple to sea. The drive or walk through the pass of Aberglaslyn is a dream of beauty, the river tumbling down its rocky bed and then sweeping out into a broad calm stream on its way to the sea at Portmadoc. Portmadoc, too, is a most picturesque little town, the busy centre of traffic for the conveyance of the slate from the Ffestiniog quarries; and just inland, joining Portmadoc, lies Tremadoc, overhung with a grandly rocky bit of mountain, and possessing a most picturesque, ivy-covered church, one of the few spired churches in North Wales.

From Portmadoc runs the little narrow-gauge railway, originally constructed for the use of the quarries, now largely used as a tourist convenience. Up it rises by repeated gradients through the woods which clothe the sides of the mountain to a height of 700 ft., the terminus being Duffws. Here the slate quarries are well worth a visit, or if scenery be preferred, the village of Ffestiniog, with its falls, is not far off. The glimpses down the Vale of Ffestiniog from the train are grandly beautiful. Delightful little walking tours might be made throughout this whole district, with comfortable and reasonable accommodation at the various hotels. For these every information is given in Baedeker's "Guide to North Wales," with which every visitor ought to be provided.

But fascinating as these various expeditions cannot fail to be to lovers of beautiful scenery, the will to make them must often outrun the power in the case of a tired nurse, for whom the truest rest does not consist in long walks, or even drives; and it is for these that Criccieth possesses such special charms. Here, if a lodging is obtained on the Marine Terrace (and would-be visitors may communicate with the Editor on this point), a perfect view of sea and mountain faces the occupants of the cosy little sitting-rooms; whole days might be profitably spent on the grassy slopes of the Castle Rock, only a stone's throw away, while delightful strolls may be had along the shore or behind the town up to the Flagstaff Hill. The Black Rock, Craig Dhu, is a mile and a-half off, the best way to which lies along the railway (which serves in Wales as a convenient high road from one place to another), and here are splendid stretches of sand with caves full of sea treasures, and grassy slopes between the rocks on the hillside which make the most delicious places for picnic meals. For those who know and care a little about geology or botany there is endless interest, for wild flowers and ferns are singularly abundant, and the hedges are mines of delight to their admirers. On Moel Hebog, which is within the reach of good walkers, is found the pretty and rare parsley fern.

On the journey to or from Wales, if the London and North-Western be the route chosen—and a very beautiful one it is by Rhyll and Bangor, along the Menai Straits, and down into the country of mountains—a stop should be made at Carnarvon to see the grand old castle, and if possible Conway, too, should be visited, with its ancient and splendid ruined stronghold.

A feature of North Wales, which is perhaps not realised until after personal experience, is the curious sensation of suddenly finding oneself among a people of another language, and other manners, too, than their neighbours of England proper. That, somehow, adds to the enjoyment of the expedition. Within a seven hours' railway journey from London, on the same island, to be met everywhere by people to whom English is almost a dead letter, and of whose tongue you comprehend not one syllable, has a delightfully unusual element in it. The Welsh have manners which are a vast improvement upon those which obtain nearer civilised centres, and their soft musical voices fall like a charm upon ears accustomed to the hard cockney twang.

To thoroughly enjoy a holiday in mountain land, clothes that weather will not hurt, and stout, nailed boots are essentials. Also skirts that well clear the ground, and hats that defy wind. Thus equipped seekers after health and enjoyment cannot do better than spend their next holiday among the Welsh mountains, should that delightful region be to them unknown land.

### Everybody's Opinion.

[Correspondence on all subjects is invited, but we cannot in any way be responsible for the opinions expressed by our correspondents. No communications can be entertained if the name and address of the correspondent is not given, or unless one side of the paper only be written on.]

### DIFFICULTIES OF A PROVINCIAL MATRON.

A "Provincial Nurse" writes: On behalf of four nurses may I thank a "Physician to a Provincial Hospital" for his letter, which was published last week? When we read the sweeping charges brought against provincial nurses by this matron we all hoped someone would write and defend the nurses. It is well known that there are two sides to every question, and as the matron has made such admirable use of her side, may I, on behalf of the nurses, air my grievances? Some little while ago we had a change of matrons here, and at first we were weak enough to hope it was a change for the better. But, alas! truly "he that lives on hope will die fasting," for we were quickly undeceived. We had hoped that having been a nurse (this being her first post as a matron) she would have a little more fellow-feeling and sympathy for us and our shortcomings; but instead of this, she seemed doubly hard, evidently her idea of ruling being to make herself eminently disagreeable, not only to the nurses but the whole of the staff, at the same time taking very good care to keep on good terms with the Visiting Committee; possessing, too, a most disagreeable habit of ordering and correcting the nurses in the wards. Of course, it is not to be supposed for a moment that they will allow this, and small wonder what conduct they may resort to—even that of a third-rate shop-girl might be preferable to this of a second-grade matron. I can fully endorse all that the "Physician" says respecting the nurses in provincial hospitals, having been in large and small ones myself, and I certainly think that nine out of ten matrons make the mistake of trying to rule by fear. Everyone knows that there is very much that is disagreeable in a nurse's life, and surely it is the matron's duty to do her utmost to make those under her charge as happy as possible. It is often quoted that poets are born and not made, and this is certainly equally true regarding matrons; it does not follow that a good nurse is a good matron—far from it, and in considering applications for the post of matron in future it would be desirable and greatly promote the happiness of patients and nurses to inquire if the applicants are capable of ruling.

### FEVER NURSING.

A "Medical Superintendent" writes: "A Looker-on," while expressing great faith in the aphorism from which he takes his *nom de plume*, has, I would venture to point out, taken hold of the wrong end of the stick. He argues that the best type of nurse will not adopt fever work as a speciality so long as responsible positions in fever hospitals are given only to those who have had general training. He regards the absence of scope for ambition, therefore, as the fundamental cause of the dearth of good fever nurses, and is satisfied that the evil would be overcome by appointing them to posts for which they are, in the case of many institutions, at present regarded as ineligible. As a matter of fact, the appointment of so many general trained nurses to responsible posts is a result, and not the cause, of the scarcity of good fever nurses. Indeed, this is implied in the letter of "A Looker-on." He states, in the first place, that the general nurse is appointed on the strength of "a sound general training," and in the second that, "all things being equal, the fever-nurse should have a preferential claim on responsible posts in fever hospitals." Now, making all allowance for personal excellence on the part of any given fever nurse, under present conditions the question of her equality with her general trained sister cannot be entertained. It is, as indicated by "A Looker-on," entirely a matter of training—and in how many institutions is it possible for the fever-nurse to receive a training, which, although specialised, is fairly comparable with the "sound" one obtainable in innumerable general hospitals? How many fever hospitals are in a position to grant a certificate as the fruit of a curriculum worthy of such a comparison? In the answer to this question lies the true root of the evil. It is little use crying out for good fever nurses until something more has been done to produce them. Surely the time has come for the general adoption by the larger fever hospitals of a definite curriculum for their nurses, including, in addition to ward instruction, the elements of anatomy, physiology, hygiene, and dispensing; the outlines of general medical and surgical nursing; and, in special detail, the pathology, clinical history, management, nursing and treatment of the various fevers. Speaking from personal experience, I am certain that the prospect of obtaining a thorough training and a certificate would attract probationers of the best type in increasingly large numbers. In this way the want of good fever nurses would soon cease to be felt, while the "new" fever nurse would have little difficulty, on her own ground, in competing with her general-trained rival.

[We are always glad to find nurses studying theory—so long, that is, as it does not interfere with practice. It must never be forgotten that the very essence of a nurse's training is practical work on living cases, and we fear that any attempt to give a general medical and surgical training in a fever hospital must depend largely upon theoretical teaching, and must therefore fail. At the same time there can be but little doubt that most nurses who are trained only in general hospitals are but imperfectly equipped in what, so far as private nursing is concerned, becomes one of their most important duties, that is, in the nursing of fever cases; and we cannot but hope that before long some form of combination will be entered into between general and fever hospitals by which the nurses trained in each will be enabled to pass through the wards of the other. "Institutes" which send nurses to be trained sometimes take care to pass them through the various branches of their work, but it is too often the case that the training of probationers at a general hospital, as at a fever hospital, is limited to the class of case that hospital receives.—Ed. *T. H.*]

### Novelties for Nurses.

A special exhibition of art linens and pure flax embroideries is being held this season at Messrs. Harris and Sons, 25, Old Bond Street. The novelties there displayed are just such as will appeal to hospital nurses, many of whom will find their way to these fascinating showrooms there, the special attraction for nurses being the delightful new linens in fast dyes—the "G G" linen they call it, at 2s. 6d. per yard, one yard wide. A clear navy blue which would set even the London laundresses and all her evil works at defiance, was also much "en evidence," and we prophesy a good sale for a pink one of a most becoming shade. Greys, light and dark, or crushed strawberry, in fact, almost every shade was represented in this charming material. There is something in linen—real pure linen—which is always attractive to women; possibly it is an inherited taste from the days when the hum of the hand-loom was heard in the land, and it was as natural for women to learn to spin as it will be for the next generation to learn to bicycle. Those of us who wear uniform will be pleased with all the dainty appointments suitable for nurses' costumes which are shown at this exhibition. The apron—a most important item—is not forgotten, and is displayed by Messrs. Harris in most attractive designs, besides being made of a really durable fabric. By the bye, the firm has arranged for the making up of their own materials; an estimate is always gladly given if asked for, when the colour, &c., may be selected. The linen in various shades of grey would be useful for district nurses, made up as coat and skirt, and as the material is ready shrunk the washing need be no matter of anxiety. It is well, too, to bear in mind that Messrs. Harris accept orders for designing and embroidering all kinds of institutional badges, hem-stitching and embroidering linens, aprons, &c.; another speciality of the firm being the supply of lovely working materials, ready traced, for nurses whose leisure moments can be given to the needle.

### A Book and its Story.

### PIERROT.*

We own to a sense of regret when the last page of this little volume is read. During its perusal we have laughed and wept, and lived and suffered with the quaint personalities herein described. And creatures of imagination though they are—to us they have seemed real, living people—with more interest about them, perhaps, than it is commonly given to an everyday mortal to possess. Their surroundings—the old world chateau, the avenues, the gardens where the Piping Faun was—they are real, too, and assuredly are not evolved out of the world of the imagination! The romance and the reality of the story has a very breath of France about it. At times grave, at others gay, we read "Pierrot" through with an interest that never flags, and are carried along by the whimsical play of the writer's unusual measure of imaginative power. But the book possesses something more than appears upon a superficial reading; it is full of suggestiveness, and thoughts which are deeper perhaps than are the actual words.

The boy Pierrot writes autobiographically in the year 1870 from the seclusion of an ancestral home, beyond the walls of which he is in ignorance of any outside world. "You must know," he begins, taking the reader at once into his confidence, "when the General went off to beat the Prussians he left behind him, here, in the Château de Pamiers—imprimis, me; secondly, Joniaux; thirdly, Lisette, to cook the food; and Pierre, the stable boy. . . Well, this morning at breakfast the post came and brought me a letter from the General. For months past he has been at law with a man called Oppenheim, a half German. They have been fighting over 6,000 francs like two dogs over a bone. The courts gave judgment for my father, but now that war is declared he wants to return the money. His letter enclosed a cheque for 6,000 francs and an order for me to go to the bankers in Paris, change it into gold, and pay it to this old Oppenheim." From this moment his life is changed, and it is not as a child he writes any more; he has all the intolerance of restraint born of youthful years.

Paris robbed the boy of his youth.

"I am half changed already," he declares, on the afternoon of his arrival in the French capital. "I have taken my father's money to buy chocolate and cream tarts and a 'Pierrot' suit. And a ticket for the ball. I feel nothing can stop me from that, for I have read about balls, and wish to see one." The General's cheque never sees its lawful owner.

Of this Opera ball the boy writes: "Someone slung me round by the arm, and I was in the middle of the dance, clasping a girl's waist; I didn't dance, but she did. I was in a whirl of flying muslin and laughter. The whole world seemed spinning like a top to the music of the band; gipsies, monkeys, bears, and bull-fighters were flying round us. A thousand footsteps keeping time to the music that seemed gone mad. The girl's hair was amber-coloured, and her eyes black; her silk dress only reached to her knees; the left half of it was amber colour, and the right black, and her stockings matched."

"The black and amber girl was a whirlwind, and like a bit of straw," Pierrot writes further of this ball. "She was the music, she was the light. We were dancing all alone in a mist. And away we whirled into a dance grown madder than before. Twice, three times, four times. Knots of violets were flung in the air, roses, camellias."

"Ambre-Noir" is her name, the boy's fellow masquerader informs him, and she calls him Pierrot; gay, lovely, and witty, little Ambre-Noir exercises a sovereignty over the senses not to be wondered at. After this, she is much with her new captive, her love for Louis de Maureval redeems her, but he assures himself he feels no love for her, no real love. Instead, he gives his affections to a creature of the spirit world—of whom the reader must read for himself—but our sympathies are less with the Day Dream love than the bewitching, fascinating, and less unearthly woman. Back again once more in the old château, Pierrot says of Ambre-Noir some days after the ball, "She came yesterday, and she was quite a different woman; for one thing, she was sad, and then she was dressed differently. We sat down among the ferns to talk. 'Why do you cry?' I asked. 'What makes you so sad? Have I done anything?' "'You? Yes; everything. You have made me love you—and other things, too. This lovely evening, the sunlight, the ferns, the birds—oh! listen to them; they will break my heart. If I could only die now, and forget Paris and the past.' "'Promise me one thing, Louis," she added after a while. 'Promise me one thing?' "'Yes.' "'That you will never ask me any questions.'"

And Louis de Maureval kept his word; nor, indeed, was it difficult for him to do so. For after this Ambre-Noir does not cross his path again for some time. But in her place there appeared to the young man the living impersonation of his dreams, a spirit form, to whom we have already alluded, and who had been ever at his side. Since the night of the ball she had deserted him. This is the fantastic, mysterious part of the book, which, to be rightly understood, must be read in the author's own words. To her, to this unearthly being, Louis de Maureval gives anew an unswerving allegiance. The gates of Paris are closed fast, and within their fastnesses is Ambre-Noir.

The siege of Paris has begun. The sky is filled with the roar of the cannon; now and again a tearing, screaming sound comes through the stillness of the deserted country-side, the firing of the mitrailleuses. France is at bay—wounded, bleeding, overwhelmed, but still fighting and tearing her enemies. So Pierrot depict his surroundings at this time. Then news comes from a fellow officer of the General's. "He was shot by a Prince, and he died a soldier," so ran the letter to the household.

Louis de Maureval succeeds to his father's title and estates. He continues his converse with his visitant from the unseen world; Ambre-Noir is remembered only as an episode out of the past. Time goes on; it is spring again. The war is over. The gates of Paris are unclosed. Ambre-Noir finds her way once more for the last time to the gray château. This time Pierrot writes of her with genuine sorrow. "She had found the château in ruins, and in the desolate hall, as she stood calling for me, she had been shot at by Joniaux, through a pitiable mistake." The girl is laid on a table in speechless misery by the master and his man. They cannot staunch the wound, they recognise her death as inevitable.

"And she went on talking, chattering, as well as she could," the story goes on in Pierrot's words; "quite delirious, seeing nothing, yet speaking to me. Telling me how she had been in Paris during the siege. She had been living with an aunt; she wanted to start a millinery business; she wanted to earn money honestly, so that she might be able to meet me without blushing, and would I promise never to ask her about the past?

"Now she was at the ball, and, suddenly raising herself on her arm, she looked me full in the face with a puzzled expression.

"'Pierrot!' She said the word, and fell back dead."

Louis de Maureval's concluding words convey many self-reproaches. "The only thing that I regret," he writes, "is the golden head of Ambre-Noir, which I shall see no more in this world or the next. If I had not stolen the money of the General I should not have met her; she would not have loved me; she would not have died. She suffered for my fault, but not so bitterly as I have suffered for her death, so perhaps the balance stands equal, and because I shall never meet her again, let my body here make its confessions. I loved her, but knew it not till she was dead."

*By H. de Vere Stacpoole. John Lane, London, 1896.

### Where to Go.

Stafford House.—A concert and dramatic performance in aid of the Clapham Home for the Dying, under the patronage of the Princess of Wales, takes place at Stafford House on June 8th, by permission of the Duchess of Sutherland, at half-past three p.m.

North Argyll Nursing Association.—A morning concert will be given at 7, St. James's Square, by permission of Lord Egerton of Tatton and the Duchess of Buckingham and Chandos, on June 8th, in aid of this association. It is under the patronage of Princess Louise, Marchioness of Lorne.

North-Eastern Hospital for Children.—A grand bazaar and rose fête in aid of this hospital will take place at the Queen's Hall, Langham Place, on June 23rd, 24th, and 25th. The Duchess of Connaught opens it on the first day at three p.m. On the two following days the bazaar will be opened at the same hour—on the 24th by the Duchess of Marlborough, on the 25th by the Duchess of Newcastle.

Royal British Nurses' Association.—We are requested by the secretary to state that the annual meeting of the Royal British Nurses' Association will be held, by kind permission of the treasurer, at the Great Hall of St. Bartholomew's Hospital, London, E.C., on Wednesday, July 22nd, at twelve (noon). Luncheon will be served in the banqueting hall, St. James's Restaurant, Regent Street, and Piccadilly, W., at half-past one p.m. Tickets, price 2s. each, can be obtained from the secretary, R.B.N.A., 17, Old Cavendish Street, London, W., on and after June 15th, by prepayment only.

### Notes and Queries.

The contents of the Editor's Letter-box have now reached such unwieldy proportions that it has become necessary to establish a hard and fast rule regarding Answers to Correspondents. In future, all questions requiring replies will continue to be answered in this column without any fee. If an answer is required by letter, a fee of half-a-crown must be enclosed with the note containing the enquiry. We are always pleased to help our numerous correspondents to the fullest extent, and we can trust them to sympathise in the overwhelming amount of writing which makes the new rules a necessity. Every communication must be accompanied by the writer's name and address, otherwise it will receive no attention.

### Queries.

(53) *Training.*—Can you tell me of any hospital where probationers are received at the age of 20?—*Belfast.*

(54) *Nursing in India.*—Can you tell me the address of the Up-Country Nursing Association in London?—*M.E.*

(55) *Indian Hospitals.*—Can you give me a list of hospitals and asylums in India?—*Enquirer.*

(56) *South Africa.*—To whom should I apply for information about hospital work in South Africa?—*Mona.*

(57) *Useful Experience.*—Will you advise me how a lady, who does not wish to become a professional nurse, but only to increase her usefulness in daily life, can obtain a short period of training in nursing? She cannot pay a premium.—*E. L. T.*

### Answers.

(53) *Training (Belfast).*—In "How to Become a Nurse" (Scientific Press, 428, Strand, W.C.) you will find a list of hospitals and training schools, with all particulars as to qualifications. Only children's hospitals take such young probationers.

(54) *Nursing in India (M.E.)*—Write to the hon. secretary, Major-Gneral J. Bonus, R.E., The Cedars, Strawberry-hill, S.W.

(55) *Indian Hospitals (Enquirer).*—In Burdett's "Hospitals and Charities" (Scientific Press, 428, Strand, W.C.) you will find a full list of hospitals in India. We are very glad you find The Hospital useful and are so constant a reader.

(56) *South Africa (Mona).*—You will find a list of South African hospitals in Burdett's "Hospitals and Charities" (Scientific Press, 428, Strand). Write direct to the matrons for the information you require.

(57) *Useful Experience (E. L. T.)*—It is doubtful if you wou'd anywhere obtain admission into a hospital for a short period without payment. At many hospitals paying probationers are received for three months; the fee is usually a guinea a week. The only course we can suggest would be to advertise.

*Small-pox at Gloucester.*—"Truth" is informed that no attention can be paid to anonymous communications. Anyone wishing a letter to appear in "Everybody's Opinion" must send with it full name and address for the Editor's information.

*Private Nursing Institutions at Margate.*—"Nurse A." is reminded that every query must be accompanied by the name and address of the writer.

### For Reading to the Sick.

### TRUSTFULNESS.

**Motto.**

Be prompt to move, but firm to wait.—*Wordsworth.*

**Verses.**

Not stirring words, nor gallant deeds alone,
Plain patient work, fulfilled that length of life,
Duty—not glory—service, not a throne,
Inspired his effort, set for him the strife. —*Clough.*

The highest duties oft are found
Lying upon the lowest ground—
In hidden and unnoticed ways,
In household works on common days.
Whate'er is done for God alone
Thy God acceptable will own.

"The time is short," this day may be
The very last assign'd to thee;
So speak that should'st thou ne'er speak more,
Thou may'st not this day's words deplore.

Am I wrong to be always so happy? This world is full of grief;
Yet there is laughter of sunshine, to see the crisp green in the leaf;
Daylight is ringing with song birds, and the brooklets are crooning by night,
And why should I make a shadow where God makes all so bright?
Earth may be wicked and weary, yet cannot I help being glad!
There is sunshine without and within me, and how should I mope and be sad?
God would not flood me with blessings, meaning me only to pine
Amid all the bounties and beauties He pours upon me and mine;
Therefore will I be grateful, and therefore will I rejoice;
My heart is singing within me! Sing on, O heart, and rejoice. —*H. F. G.*

I ask Thee for a thoughtful love,
Though constant watching wise,
To meet the glad with joyful smiles,
And to wipe the weeping eyes;
And a heart at leisure from itself
To soothe and sympathise. —*A. T. M.*

**Reading.**

"We must be childlike enough to trust our Father . . . as well with His refusals as His gifts, His silence as His speech. What need to scrutinise or understand His ways? It suffices that they are His, and we are sure that all is well; that love is there, and the fruit of love not far away."—*E. F. Russell.*

"There is no true rest of soul and heart in time of trial until we have come to look beyond second causes and human instruments, and have seen the hand of our God and Father appointing *all* in His infinite wisdom and love. Cease as far as possible from vain regrets, and leave all calmly to Him, Who has promised that all things shall work together for good. To bring our hearts to this may be the very meaning and end of the dispensation."—*Anon.*

"God's design is to bring us happily to Himself in another world, and He will leave no means unessayed for this purpose. If we have the same end in view and look up to Him as carrying it on steadily for us, we may be happy both here and hereafter; if we have not, the consequence must necessarily be despondency, vexation, and fretfulness at the ways of Providence."—*T. Adams.*

## Figures and Tables

**Fig.17 f1:**
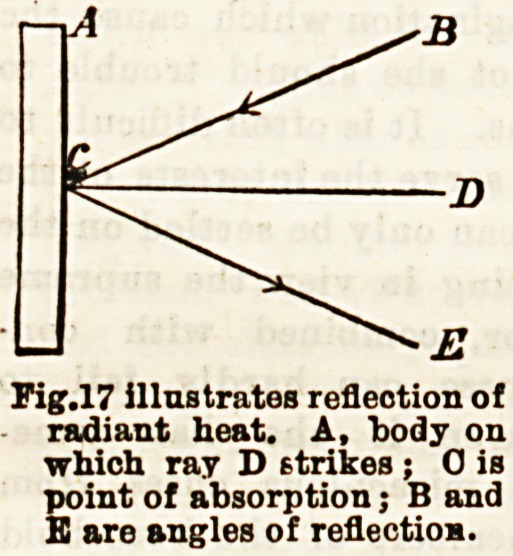


**Fig. 18. f2:**
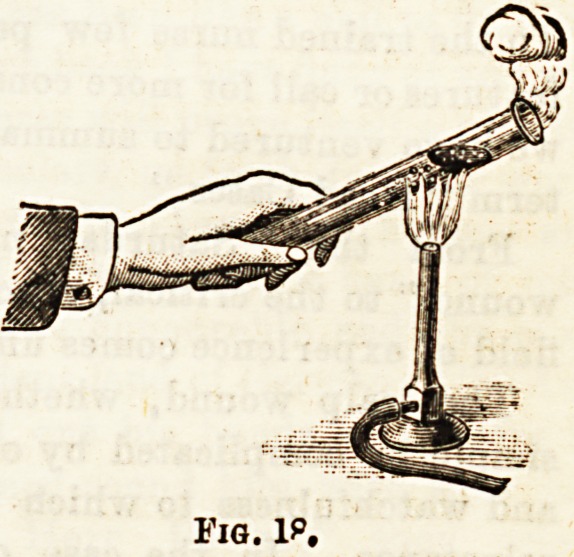


**Fig. 19. f3:**